# T Cells in Adipose Tissue in Aging

**DOI:** 10.3389/fimmu.2018.02945

**Published:** 2018-12-12

**Authors:** Antu Kalathookunnel Antony, Zeqin Lian, Huaizhu Wu

**Affiliations:** ^1^Department of Medicine, Baylor College of Medicine, Houston, TX, United States; ^2^Department of Pediatrics, Baylor College of Medicine, Houston, TX, United States

**Keywords:** adipose tissue, inflammation, T cells, insulin resistance, aging, obesity

## Abstract

Similar to obesity, aging is associated with visceral adiposity and insulin resistance. Inflammation in adipose tissue, mainly evidenced by increased accumulation and proinflammatory polarization of T cells and macrophages, has been well-documented in obesity and may contribute to the associated metabolic dysfunctions including insulin resistance. Studies show that increased inflammation, including inflammation in adipose tissue, also occurs in aging, so-called “inflamm-aging.” Aging-associated inflammation in adipose tissue has some similarities but also differences compared to obesity-related inflammation. In particular, conventional T cells are elevated in adipose tissue in both obesity and aging and have been implicated in metabolic functions in obesity. However, the changes and also possibly functions of regulatory T cells (Treg) in adipose tissue are different in aging and obesity. In this review, we will summarize recent advances in research on the changes of these immune cells in adipose tissue with aging and obesity and discuss their possible contributions to metabolism and the potential of these immune cells as novel therapeutic targets for prevention and treatment of metabolic diseases associated with aging or obesity.

## Overview

The rapidly increasing elderly population worldwide can cause a wide range of implications in healthcare policies of all nations. In the United States, the population aged 65 and over is projected to be 83.7 million by 2050, almost double the estimate of 43.1 million in 2012 (1). Both aging and obesity are associated with low-grade, chronic inflammation that may be detrimental to health. While obesity is triggered by excessive nutrient intake and sedentary lifestyle, aging is caused by deteriorative changes in adult organisms with advancing age. In certain circumstances, aging is termed “inflamm-aging,” whereas increased inflammation of adipose tissue in obesity is described as “meta-flammation” or metabolically activated inflammation (2, 3).

## Development of “Inflamm-Aging”

Aging is an intricate, dynamic, and physiologic process that adversely affects most body functions, including the development and maintenance of the immune system (4). Basic aging mechanisms such as cellular senescence and diminished number or dysfunction of immune progenitor cells are causative factors of development of low-grade inflammation (5). Immunosenescence is a term to describe the decline of immune function associated with aging, which can lead to increased susceptibility to infections, cancer, and metabolic and autoimmune disorders (6, 7).

During the state of infection or tissue damage in healthy young individuals, the innate immune system, including neutrophils, monocytes, and natural killer (NK) cells, responds quickly. In addition, the adaptive immune system is activated by the action of antigen-presenting cells (APCs), and effector T and B lymphocytes are developed and fight against the insult with a refined antigen-specific immune response. After the effective removal of the invading pathogen, the host immune response must be deactivated and return to a quiescent state to prevent further tissue damage. A subset of T lymphocytes called regulatory T cells (Treg) are responsible for suppressing the deleterious effects of immune response (6). In general, both innate and adaptive immune systems are affected by aging, but adaptive immunity, especially T lymphocytes, are most susceptible to the detrimental effects of aging. The thymus is the key organ orchestrating production of new T lymphocytes, but age-associated chronic involution of the thymus results in a reduced proportion of naïve to memory T cells in the periphery (6, 8). As the number of naïve CD8+ T cells declines with aging, the diversity of naïve and memory T cell receptors (TCRs) is also reduced significantly in mice and humans (9). In parallel, aging represents a striking decline in humoral and cell-mediated responses mainly caused by the senescence of T lymphocytes (10). Hence, gradual deterioration of the immune system over the course of time leads to a mismatch between proinflammatory and anti-inflammatory signals that may disrupt inflammatory homeostasis causing “inflamm-aging.”

## Adipose Tissue Inflammation in Aging

Aging is commonly accompanied by obesity, especially abdominal/visceral adiposity that leads to numerous health problems such as insulin resistance, metabolic syndrome, cardiovascular disease, and disability (11, 12). Adipose tissue functions as the connecting link among nutrition, metabolism, thermoregulation and proper immune system function in healthy individuals (13). Alterations in adipose tissue are major contributors to age-associated metabolic dysfunctions and other health issues (5, 14, 15).There are two types of adipose tissue depots: (1) brown adipose tissue, composed of brown adipocytes, which contain numerous mitochondria and lipid droplets and function as the site of adaptive thermogenesis (16); (2) white adipose tissue, which includes visceral adipose depots and subcutaneous adipose depots and acts as the prime location where metabolic energy is stored in the form of triglycerides during periods of nutritional excess (17). In healthy young individuals, subcutaneous depots act as a metabolic sink where all the excess calories are stored in adipocytes in the form of triglycerides (18). But after the middle age, the ability of subcutaneous fat depots to store lipids declines (19), leading to relocation of excess fat to visceral fat depots, causing visceral adiposity (20). This excessive lipid accumulation in visceral fat depots, along with the surrounding tissue microenvironment, may drive adipose tissue inflammation. Indeed, when compared to subcutaneous adipose tissue, visceral adipose tissue contains more immune cells and plays a more critical role in immunometabolic homeostasis (21). The main immune cell types in visceral adipose tissue include macrophages (ATMs) and T lymphocytes, and other immune cell types which may change in numbers and phenotypes in aging and obesity (22–29). In this review, we focus on changes in T lymphocytes in adipose tissue in aging and the potential roles of adipose tissue T cells in metabolic functions.

## Changes in Adipose Tissue T Cells in Aging

The changes in adipose tissue T cells in obesity have been well-documented in mice and humans (24, 30–33). In contrast, information on age-related changes in adipose tissue T cells is limited. Most studies showing the effects of aging on T cells has focused on lymphoid tissues and blood. It has been well-recognized that aging in humans and mice increases the proportion of memory T cells in blood and lymphoid organs (34, 35). Further, T cells in aging tend to polarize to a proinflammatory phenotype, secreting high levels of type 1 cytokines such as IFN-γ, TNF-α, and IL-6 (34, 36–39) and expressing elevated levels of chemokine receptors with enhanced chemotaxis to chemokines (40–42). In humans, one study showed that peripheral blood CD8+ T cells that are positive for IFN-γ, IL-2, and TNF-α are significantly increased with age among all three CD8+ subsets, i.e., naïve, effector/cytotoxic, and memory T cells (38). Another study revealed that intracellular TNF-α and IL-6 levels in blood T cells were significantly increased in the older age (37). This aging-associated elevation of proinflammatory cytokines could be one of the reasons for thymic involution and the reduced proportion of naïve to memory T cells (8). Thymic involution and immune system aging could result in alterations of T cell development, activation, homeostasis, and trafficking in peripheral tissues.

**Figure 1:**
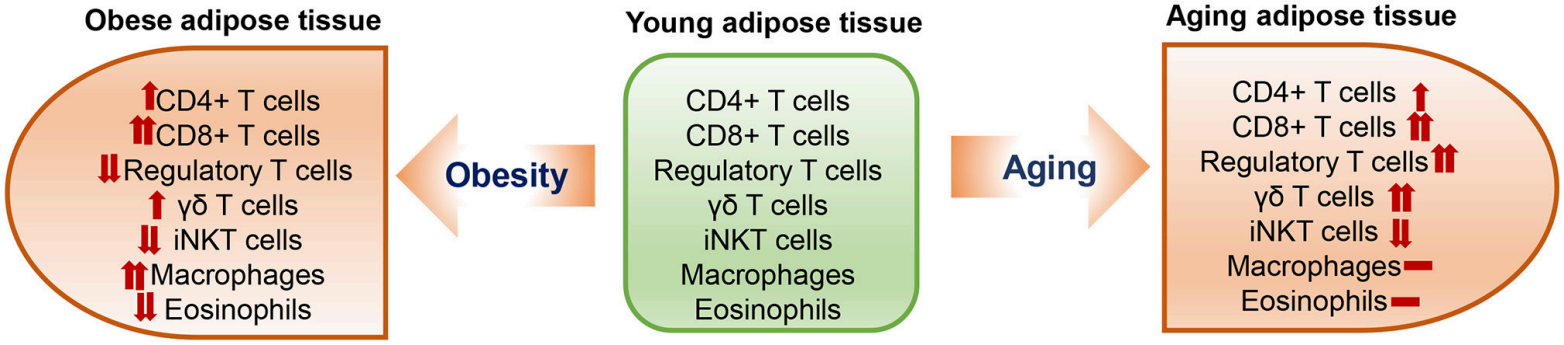
Changes in adipose tissue T cells, macrophages and eosinophils in obesity and aging.

Limited numbers of studies have shown T cell changes in aging adipose tissue; data were generated mainly from mouse models at different ages [from 10 to 15 months [“middle age”] to >18 months [“old age”]]. Aging is commonly associated with visceral obesity and has some similarities but also differences in changes of adipose tissue immune cells, including T cells, when compared to diet-induced obesity (Figure 1).

### Conventional CD4+ T Cells and CD8+ T Cells

Similar to obesity, aging is associated with significant increases in adipose tissue T cells. Compared to young mice, aged mice (18–22 months old) have ~2-fold increases in CD3+ T cells in adipose tissue when normalized to tissue weight (43). Both conventional CD4+ T cells and CD8+ T cells are significantly increased, with a greater increase in CD8+ than CD4+ T cells, in visceral fat of aged mice compared to that of young mice (43), which is also similar to the change with obesity (31, 44). The increases in adipose tissue T cells, particularly CD8+ T cells, were also observed in 11- to 16-months-old middle-aged mice (23, 45) and appeared to be influenced by sex, with females having a higher percentage of CD8+ T cells than males (45), in contrast with obesity, in which a greater change in adipose tissue T cells occurred in males than females (24). Furthermore, CD69+-activated and IFN-γ-expressing CD8+ T cells and activated CD4+ T cells are increased in adipose tissue of middle-aged mice (23, 45). These changes are similar to those in obesity (31, 44). However, the age-associated changes in adipose tissue T cells appear to be independent of adiposity (46). The increases in adipose tissue T cells in aging are tissue specific and are not observed in blood or lymphoid organs (43, 45).

### Regulatory T Cells (Treg)

Treg normally represent a small portion of CD4+ T cells, and regulate inflammation and prevent autoimmune response mainly by suppressing conventional T cell proliferation and activation (47–49). Compared to lymphoid tissues and blood, adipose tissue is highly enriched with Treg in normal conditions. When compared to their spleen and lymph node counterparts, adipose Treg are a unique population having a specific antigen repertoire and a different transcript profile (30), with overexpression of transcripts encoding transcription factors (e.g., peroxisome proliferator–activated receptor (Ppar)–γ, Gata-3), chemokines or their receptors (e.g., CCR1, CCR2), cytokines or their receptors (e.g., IL10, IL1rl1), and proteins important in lipid metabolism (e.g., Dgat1, Pcyt1a) (50). In diet-induced obesity, adipose tissue Treg are dramatically reduced (30), and this Treg reduction is accompanied by loss of the adipose Treg signature in the remaining Treg population (50). In contrast to obesity, in aging, Treg are elevated in adipose tissue and continuously rise from young age to adult, middle-age and old age in mice (23, 30, 43, 51). Compared to young mice, middle-aged to old mice have 7–11-fold increases in adipose tissue Treg, which account for >50% of total CD4+ T cells in adipose tissue (23, 43). When compared to those in young mice, Treg of aged mice (25 weeks) have substantial increases in a set of transcripts (Ppar-γ, Gata-3, Klrg1, Ccr2, and l1rl1), which continue to increase with aging and may result in local adaptation to the lipophilic, hypoxic adipose tissue environment (50).

### Other Immune Cells

In addition to adaptive T lymphocytes, innate T lymphocytes such as γδT cells and invariant natural killer T (iNKT) cells are also located in adipose tissue. Both γδT cells and iNKT cells are resident cells in adipose tissue. While γδT cells tend to be increased (52), iNKT cells are decreased in adipose tissue in obesity (53–55). Similarly, a recent study showed that adipose tissue γδT cells also increased with age in mice from 5 to 28 weeks, whereas adipose tissue iNKT cells decreased significantly with age (51).

It is also worth notice that aging is different from obesity in changes of some other important immune cells in adipose tissue. For example, it is well recognized that macrophages are increased and eosinophils are decreased in obese adipose tissue (25–28, 56). However, available data indicate that the numbers of adipose tissue macrophages and eosinophils show no or only modest changes in aging (23, 43, 46). Nevertheless, adipose tissue macrophages appear to have proinflammatory phenotypes in old mice (43).

## Functions of Adipose Tissue T Cells

**Figure 2:**
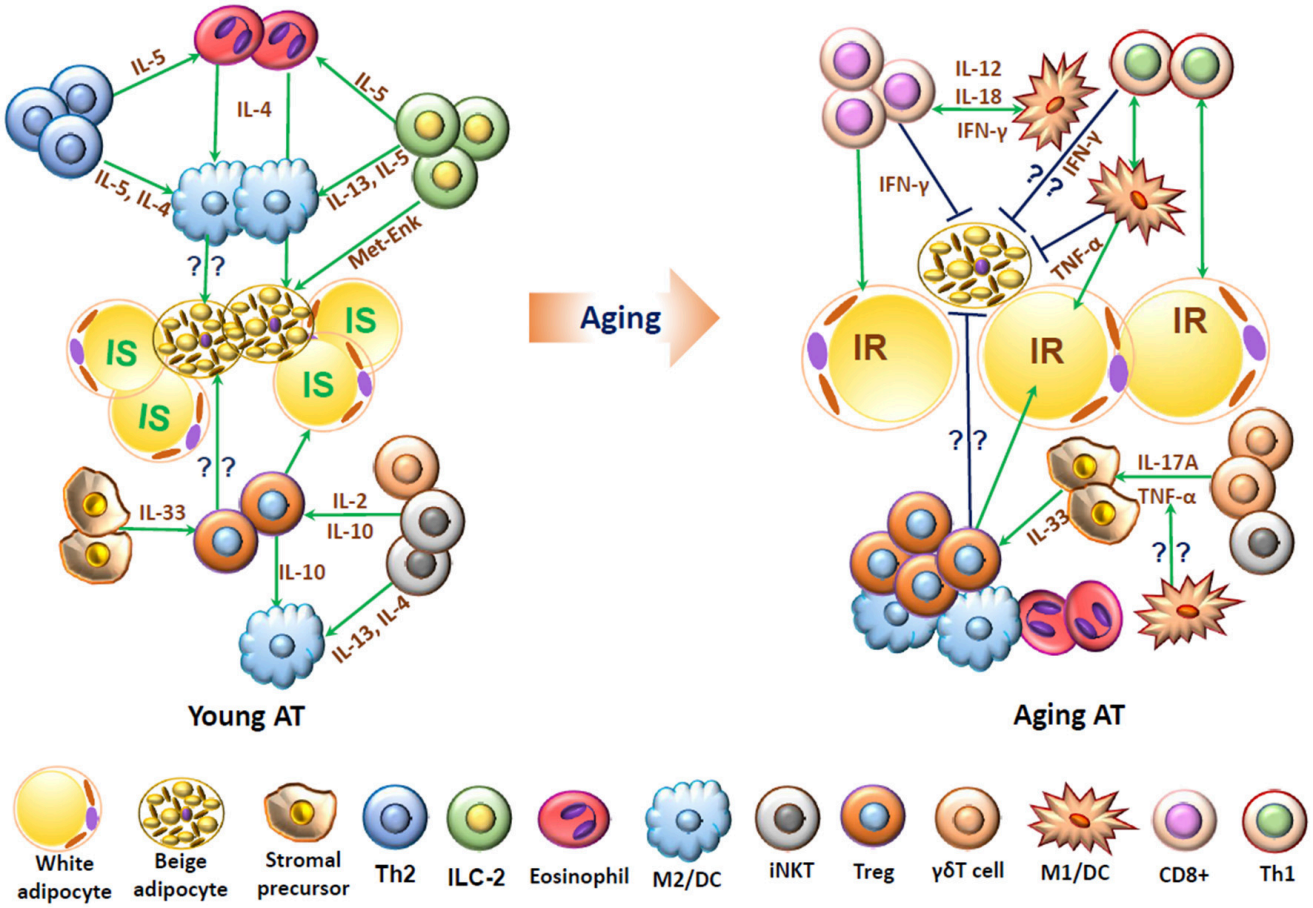
Schematic representation of functions of T cells and associated immune cells in young and aging adipose tissue. In young adipose tissue, insulin-sensitive (IS) white adipocytes, beige adipocytes, and stromal cells are surrounded by “type 2” immune cells, including alternatively activated macrophages (M2), T helper type 2 (Th2) cells, eosinophils, innate lymphoid type 2 (ILC2) cells, regulatory T cells (Treg), and invariant natural killer T (iNKT) cells, which interact with each other and produce type 2 cytokines such as IL-4, IL-5, and IL-13 and may help maintain normal adipose functions, including adipocyte insulin sensitivity and beige fat thermogenesis. In aging, adipose tissue contains increased numbers of T cells including conventional CD4+ cells, CD8+ T cells and Treg and also proinflammatory M1-like macrophages/dendritic cells (DCs), which produce proinflammatory molecules such as IFN-γ and TNF-α and may contribute to adipose dysfunctions such as insulin resistance (IR) and impaired beige fat thermogenesis.

In healthy young humans and mice, various T cell subpopulations harboring in adipose tissue may play pivotal roles in homeostasis and maintenance of immune cells, energy metabolism, and thermogenesis. Changes in adipose T cells in aging and obesity may contribute to adipose tissue inflammation and associated metabolic dysfunctions (Figure 2).

### Role of T Cells in Adipose Tissue Inflammation

Conventional T cells, including CD4+ Th1 cells and effector CD8+ T cells, are elevated in adipose tissue and may play important roles in adipose tissue inflammation in both aging and obesity (31, 43–46). Conventional T cells are important inflammatory components producing high levels of inflammatory molecules such as IFN-γ, thereby contributing to inflammation. In addition, altered T cells and related inflammatory molecules may contribute to aging- or obesity-related adipose inflammation by influencing other immune cells such as macrophages in adipose tissue (31, 43, 44, 46). Some reports showed that in obesity induced by high-fat diet (HFD), conventional T cell infiltration and accumulation are the primary events and play important roles in the initiation of adipose tissue inflammation and in ATM infiltration and activation (31, 57, 58). Indeed, combined CD4+ and CD8+ T cell deficiency in obese mice decreased ATMs and reduced adipose tissue inflammation (59). In addition, γδT cells, Vγ4, and Vγ6 subsets in particular, may also contribute to macrophage accumulation and inflammation in adipose tissue in obesity. Deletion of γδT cells or Vγ4/6 prevents obesity-induced macrophage accumulation and inflammation in mice (52). In contrast to conventional CD4+, CD8+ T cells, and γδT cells, Treg are dramatically decreased in adipose tissue in obesity, and expansion of Treg in obese mice protects against adipose tissue inflammation, with decreased ATMs and related inflammatory markers (30, 60). Conversely, depletion of Treg in young mice may increase adipose tissue levels of several inflammatory markers (30). These data suggest a protective role of Treg in obesity-induced adipose tissue inflammation. In contrast, Treg are increased in adipose tissue of aging mice, and depletion of adipose tissue Treg did not significantly enhance systemic and tissue inflammation in aging mice (23).

iNKT cells are enriched in adipose tissue and may play a role in adipose tissue Treg homeostasis by producing IL-2 in young mice (see section Regulatory T Cell Maintenance in Adipose Tissue). However, data are not consistent about the roles of iNKT in adipose tissue inflammation and insulin resistance associated with obesity, which were recently discussed in other review articles (61–63) and are not included in this review.

In addition to their crucial role in visceral adipose inflammation, T cells, conventional CD4+ and CD8+ T cells in particular, also infiltrate into skeletal muscle, mainly localized within intermyocellular and perimuscular adipose tissue, and play substantial roles in skeletal muscle inflammation in obesity (59, 64, 65). Their potential role in aging-related inflammation in skeletal muscle remains to be investigated.

Inflammation has been involved in adipose tissue remodeling (28). In particular, proinflammatory M1-like macrophages have been implicated in adipose tissue remodeling associated with obesity (66). Given the crucial roles of T cells, especially Th1 cells and cytokine IFN-γ, in macrophage M1 polarization(66, 67), T cells may also play a role in adipose tissue remodeling via regulation of macrophage phenotypes. However, a potential direct role of T cells in adipose tissue remodeling, particularly in relation to aging, remains to be studied.

### Roles of Conventional T Cells in Insulin Resistance

Inflammation in adipose tissue has been implicated in insulin resistance and metabolic dysfunctions associated with obesity. Depletion of CD8+ T cells ameliorated systemic insulin resistance, while adoptive transfer of CD8+ T cells aggravated insulin resistance in obese mice, demonstrating a crucial role of CD8+ T cells in systemic metabolic dysfunctions in obesity (31). CD4+ Th1 cells may have similar contributions to obesity-related insulin resistance; reductions in adipose tissue Th1 by ablation of major histocompatibility complex (MHC) class II molecule (MHCII) on adipocytes or ATMs were associated with improved insulin resistance in obese mice (58, 68, 69). Our study showed that combined deficiency of CD4+ and CD8+ T cells in obese mice, with reduced inflammatory status, improved insulin resistance systemically and in adipose tissue as well as in skeletal muscle (59, 65). The mechanisms underlying contributions of proinflammatory T cells (mainly CD4+ Th1 and effector CD8+ T cells) to insulin resistance may include direct adverse effects of these T cells or T cell cytokines such as IFN-γ on metabolic functions and insulin sensitivity in adipocytes or skeletal muscle through the JAK/STAT1 pathway (24, 65, 70) and T cell effects on other immune cells such as macrophages, which also play important roles in metabolic functions including insulin resistance. In contrast to Th1 and effector CD8+ T cells, Th2 cells, which produce type 2 cytokines such as IL-4 and IL-5, may protect against obesity and related insulin resistance; transfer of CD4+ T cells reverses weight gain and insulin resistance in HFD-fed lymphocyte-free mice, mainly through polarization into Th2 cells (32).

Aging is commonly associated with insulin resistance and increased prevalence of metabolic syndrome in most populations. Given the massive increases of CD8+ T cells and conventional CD4+ T cells in aging adipose tissue (43) and the discussed roles of T cells in metabolic functions, it is plausible that adipose tissue T cells may also contribute to age-related metabolic dysfunction and insulin resistance (5, 9). However, more elaborate studies are needed to unveil aging-related changes in the phenotypes of adipose tissue T cells and the exact roles of adipose tissue T cells in age-associated metabolic functions.

### Role of Treg in Metabolic Function

Visceral adipose tissue of lean mice contains more Treg cells than that of obese counterparts (30). Possible functions of Treg in lean adipose include monitoring the activity of conventional T cells and regulating proper functioning of neighboring macrophages and adipocytes (30). Gain-of-function and loss-of-function approaches demonstrated that Treg play a protective role in insulin sensitivity and energy homeostasis in obesity (30, 60). Treg may improve insulin sensitivity through the release of anti-inflammatory molecules such as IL-10 and TGF-β that may counteract the proinflammatory signals in both humans and rodents (71, 72). Consistent with these findings, Deng et al. also showed that maintenance of adipose Treg in obese mice with adipocyte-specific deletion of MHCII was associated with improved insulin resistance (69). In contrast to obesity, in aging adipose tissue, Treg undergoes significant expansion. Depletion of adipose Treg was found to be protective against age-associated metabolic dysregulation. Aging mice with depletion of adipose Treg exhibited increased insulin sensitivity compared to control mice (23), suggesting that Treg in adipose tissue may play a detrimental role in age-associated insulin resistance. The inflammatory status of mice with adipose Treg depletion did not change significantly compared to control mice. Although the mechanisms whereby adipose Treg contribute to age-related insulin resistance remain to be investigated, it seems likely that the pathophysiological mechanisms that regulate age-associated insulin resistance and obesity-induced insulin resistance may be different (23, 30).

### Roles of T Cells in Thermogenesis

Adipose tissue is one of the key organs responsible for whole body energy homeostasis via energy storage/dissipation depending on nutrient intake and external temperature fluctuations (73). Beige adipocytes, which may develop within white adipose depots, particularly in subcutaneous adipose depots, have a similar energy dissipation function as that of brown adipocytes, which is mainly induced by cold and beta-adrenergic activation (74–76). Innate and adaptive immune system components are reported to contribute to and regulate the energy storage/dissipation functions of adipose tissue (77–79). Recent reports suggest that along with macrophages, T cells may play a significant role in the regulation and maintenance of thermogenesis and overall adipose tissue energy homeostasis.

Th2 cells and associated type 2 immune cell populations such as innate lymphoid type 2 (ILC2) cells and eosinophils in young lean adipose tissue have a significant role in defining a favorable adipose niche for beige adipocyte development and thermogenesis (80). The major cytokines produced by Th2 cells, eosinophils, and ILC2 cells during type 2 immune response are IL-4, IL-5, and IL-13 (81, 82), which may promote the proliferation and differentiation of PDGFRα+ adipose stromal precursor cells to thermogenic beige adipocytes and therefore help to maintain thermogenesis in young lean conditions (83). In addition, type 2 cytokines and eosinophils are essential factors for the differentiation and propagation of alternatively activated M2 macrophages (56), which may play a role in inducing adipose thermogenesis by producing catecholamine (77). However, a more recent study by Fischer et al did not show a role of M2 macrophages in inducing adipose thermogenesis (84).

In addition to Th2 cells, PLZF+ γδT cells, and iNKT cells may also contribute to induction of adipose thermogenic function (51, 85, 86). Adipose-residing γδT cells are important for the preservation of body temperature and thermogenic function, possibly by producing IL-17A. Under cold challenge, mice deficient in either γδT cells or IL17A have reduced UCP-1 expression and are unable to survive (51). In obesity, activation of iNKT cells induces adipose thermogenesis, leading to weight loss, in mice, likely through induction of FGF21 (86). The action of FGF21 in white adipose tissue is implicated by the activation of PGC1α along with induction of adiponectin, resulting in improved energy expenditure (87, 88).

In aging, the functional beige adipocytes decline, as the number of fully active beige adipocytes in human and mice depend on the whole body metabolic fitness (89–91). How changes in adipose tissue immune cells contribute to age-related decline in adipose thermogenic functions remain largely unknown. Depletion of adipose Treg in mice reduced age-associated weight gain and adiposity, with enhanced energy expenditure (23), indicating a role of adipose Treg in age-related energy metabolism. Recently, Moysidou et al. demonstrated an inhibitory effect of CD8+ T cells on adipose thermogenesis in mice, possibly by secreting IFN-γ, which may have direct effects on thermogenesis or interfere with the effects of other immune cells, such as eosinophils and ILC2 cells, on thermogenesis (79). It is possible that conventional CD4+ T cells, particularly Th1 cells, have similar functions in adipose thermogenesis because of IFN-γ expression (79). Based on the elevations in CD8+ and conventional CD4+ T cells in aging adipose tissue, it is reasonable to hypothesize that these immune cells may play a role in age-associated decline in adipose thermogenic functions and energy expenditure. However, this hypothesis remains to be tested.

## Mechanisms for Changes in Adipose Tissue T Cells

### Regulatory T Cell Maintenance in Adipose Tissue

Treg are resident cells in adipose tissue (30). These adipose Treg in mice are seeded from the thymus during an early stage of life and expand within adipose with aging (23, 43, 92). Recently, Li et al. showed that immature Treg from the thymus undergo a priming step in the spleen prior to infiltration into adipose tissue, which may permit them to leave lymphoid organs and to survive in non-lymphoid organs, including adipose tissue (93). While iNKT cells, a type of lipid-sensing innate T cells, may assist in regulating adipose tissue Treg number and function in young mice by producing IL-2 (85), two factors, i.e., interaction of TCR-antigen-MHCII on APCs and cytokines such as IL-33, may be the main drivers of visceral adipose Treg accumulation in aging (92). Treg in aging adipose tissue express high levels of ST2, a receptor for IL-33, and IL-33 efficiently induces Treg differentiation and expansion in aging visceral adipose tissue (23, 93, 94). Recently, a subpopulation of γδT cells termed PLZF+ γδT cells was demonstrated to play a considerable role in age-related adipose Treg accumulation via producing IL-17A, which induces stromal cell production of IL-33 in adipose tissue (51). Two subpopulations of APCs have been identified in aging mouse visceral adipose tissue—MHCII+CD11b+CD11c+ macrophages and MHCII+CD11b-CD11c+ dendritic cells—both of which were colocalized with Tregs and may play important roles in Treg maintenance within adipose tissue in aging, possibly via the TCR-antigen-MHCII interaction (92). Importantly, PPAR-γ, the master regulator of adipocyte differentiation, has been shown to be a crucial molecular driver for Treg cell accumulation and function in visceral adipose tissue (95). In obesity, Th1 inflammation mediated by obese adipocyte- or macrophage-expressed MHCII may contribute to the reduction in adipose Treg in diet-induced obesity via producing IFN-γ, which blocks the effects of IL-33 on Treg proliferation. Adipocyte-specific deletion of MHCII prevents diet-induced adipose inflammation and Treg reduction (69). In addition, influx of inflammatory macrophages, release of inflammatory cytokines and imbalance of adipokines in obesity may restrict the survival of adipose tissue Treg by modulation of the adipose tissue microenvironment (30, 67, 96).

### Conventional T Cell Infiltration Into Adipose Tissue

Infiltration or migration of T cells into lymphoid organs or peripheral tissues is tightly and specifically regulated by collective effects of various adhesion molecules and chemokines/receptors (97–99). Several reports explained the mechanisms for infiltration of conventional T cells into adipose tissue and the roles of adhesion molecules and chemokines/receptors under obese conditions, but few reports are available for those related to aging. Using mouse models of obesity, our group observed that CD11a, a β2 integrin that is highly expressed on T cells, is upregulated in obesity and plays a crucial role in CD8+ T cell infiltration in adipose tissue in obese mice (44). In addition, dysfunctional, damaged or necrotic adipocytes and immune cells, including T cells, can secrete chemokines that may accelerate lymphocyte homing into adipose tissue (100). In our earlier study, we observed that RANTES, a CC chemokine (CCL5), and its receptor, CCR5, were upregulated in adipose tissue of obese mice and humans and that RANTES was colocalized with T cells within mouse adipose tissue. Our *ex vivo*/*in vitro* studies indicated that RANTES is an adipokine that can be produced by adipocytes and plays an important role in T cell migration, suggesting a potential role of the RANTES/CCR5 axis in adipose T cell accumulation in obesity (24). Another report showed that the preadipocyte- and endothelial cell-derived stromal-derived factor-1α (CXCL12), mediated early infiltration of CD4+ T lymphocytes in obesity, which preceded the increase of macrophages in adipose tissue of mice on HFD (101). In obese humans, adipocyte-secreted CCL20 may contribute to the accumulation of CD4+ helper and CD8+ cytotoxic T lymphocytes within adipose tissue, possibly via interaction with CCR6 that was upregulated on T cells in obese adipose tissue (100). However, the key molecules that mediate T cell infiltration into adipose tissue in aging remain to be identified.

### Activation of Conventional T Cells in Adipose Tissue

#### CD4+ T Cell Activation

TCRs identify the presence of a specific antigen by binding to short peptide sequences from the antigen that is displayed on APCs. These short peptide sequences from the antigen are usually presented on the cell surface of APCs with the help of MHCII molecules, which are crucial for activation of CD4+ T cells (102). Classically, naïve CD4+ T cells become activated and differentiated to effector T cells by three signals: signal 1, interaction of TCR with a peptide antigen-MHCII complex carried by APCs; signal 2, costimulatory signals such as CD28 and cytotoxic T lymphocyte antigen (CTLA) expressed on T lymphocytes and their ligands CD80 and CD86 expressed on APCs; and signal 3, cytokines such as IL-12, TGF-β, and IL-10 secreted by APCs and Treg (29, 58). Deng et al. reported that both visceral and subcutaneous adipocytes from obese humans and mice expressed all MHCII components required for antigen presentation and increased levels of CD80 and CD86, and may therefore function as APCs. Indeed, the primary adipocytes isolated from obese mice could induce antigen-specific CD4+ T cell activation (58). Xiao et al. further described that mostly large adipocytes from obese adipose tissue exhibited an elevated expression level of MHCII molecules and acted as APCs to activate CD4+ T cells to secrete IFN-γ (103). In the early stage of obesity induced by HFD, elevated free fatty acids may be the initial stimulus for adipocyte hypertrophy and MHCII-related gene upregulation, possibly via activation of JNK and STAT1, which may further activate CIITA, a prime regulator of MHCII expression (103, 104). As obesity progresses, free fatty acids may act synergistically with IFN-γ to upregulate MHCII on adipocytes. Studies by Morris and Cho et al. indicated that ATMs colocalized with T cells in lymphoid clusters within adipose tissue and may act as APCs, which express high levels of MHCII and also costimulatory molecules and process and present antigens to induce CD4+ T-cell proliferation and activation in adipose tissue of obese mice (29, 68, 105). Taken together, one important mechanism for obese adipose CD4+ T cell activation may be mediated through MHCII expressed on ATMs and adipocytes. However, its role in aging-related adipose tissue CD4+ T cell activation remains to be investigated.

#### CD8+ T Cell Activation

Compared to CD4+ T cells, CD8+ T cells show a greater increase in adipose tissue in obesity and in aging (31, 43, 106). Similar to CD4+ T cells, CD8+ T cells exhibit effector memory or effector phenotypes expressing elevated levels of IFN-γ in obese adipose tissue (31, 44). The mechanism for CD8+ T cell activation in adipose tissue is not fully understood. Nishimura et al. showed that adipose tissue from obese mice induced proliferation of splenic CD8+ T cells, indicating a CD8+ T cell-activating environment in obese adipose tissue (31). In addition to a role in adaptive immunity, memory CD8+ T cells are involved in innate immunity, being able to become activated and to proliferate under cytokine stimulation (107, 108). Indeed, CD8+ T cells from mouse adipose tissue respond to cytokines and become activated and proliferate under stimulation of IL-12 and IL-18, which are mainly produced by APCs and are elevated in obese adipose tissue (44). Results from a CD11a-knockout mouse model revealed that CD11a also plays a pivotal role in adipose CD8+ T cell trafficking, proliferation, accumulation and activation (44).

In parallel to the changes in adipose CD8+ T cells in obesity, aging is reported to accelerate accumulation of CD8+ T cells in adipose tissue, which may contribute to increased adipose inflammation. However, the mechanisms for the aging-related changes in adipose tissue CD8+ T cells remain unknown. From the above discussion regarding the impact of immune system aging on T cell homeostasis and phenotypes in lymphoid organs and peripheral blood, it is reasonable to hypothesize that immune system aging may contribute to the changes in adipose tissue T cells and inflammation associated with age.

## Conclusions and Perspectives

Similar to obesity, aging is associated with visceral adiposity and metabolic dysfunctions, including insulin resistance. Numerous studies have investigated the potential mechanisms and functions of various subpopulations of adipose T cells in obesity and related metabolic complications. Limited reports have also shown expansion of T cells, including conventional T cells and Treg, in adipose tissue in aging. However, little is known about the mechanisms of adipose T cell accumulation and their role in metabolic diseases associated with aging. Hence, future studies will need to address mechanisms and functions of adipose T cell populations in aging. In particular, some key questions need to be addressed. First, do the changes in adipose tissue T cells observed in aging mice also occur in humans? Second, what are the major factors that drive accumulation and phenotypes of various types of T cells in adipose tissue in aging? Third, why do adipose Treg function differently in age- and obesity-associated insulin resistance? Fourth, how do other T cell subpopulations, conventional T cell populations in particular, contribute to age-related metabolic disease? Finally, and most importantly, will targeting immune cells and inflammation be practical and beneficial in preventing and treating age-related metabolic disease?

In recent years, some clinical trials have illustrated the potential of targeting inflammation with pharmacological agents to treat metabolic diseases. Improvements of glucose metabolism and β-cell function and reduction of HbA1c were reported in diabetic patients after treatment with anakinra, a recombinant analog for IL-1Ra that blocks the action of the inflammatory cytokine IL-1β (109, 110). In another study, a selective JAK1/JAK2 inhibitor, baricitinib, was found to be effective in treating diabetic kidney disease and also lowering HbA1c in patients with type 2 diabetes and diabetic nephropathy (111, 112). Cenicriviroc, an oral dual chemokine receptor CCR2/CCR5 antagonist, was recently shown to ameliorate insulin resistance, hepatic inflammation and fibrosis in obese humans and mice with non-alcoholic steatohepatitis (113, 114). However, to date, inflammation-targeting therapies have not been very successful in treating metabolic diseases, particularly in humans. Further, because of the chronic nature of most metabolic diseases, the potential side effects (vs. benefits) of long-term use of inflammation-targeting drugs need to be evaluated. Nevertheless, further advances in our understanding of the roles and mechanisms of inflammation in metabolic diseases may open up novel avenues for the discovery of newer classes of pharmacological targets/agents for diabetes treatment, which may also provide novel opportunities for prevention and treatment of age-associated metaboli disease.

## Author Contributions

HW contributed to manuscript initiation and revision. AK and ZL contributed to manuscript writing.

### Conflict of Interest Statement

The authors declare that the research was conducted in the absence of any commercial or financial relationships that could be construed as a potential conflict of interest.

## References

[1] Ortman JM Velkoff VA Hogan H An Aging Nation:The Older Population in the United States. Current Population Reports -U.S. Census Bureau P25-1140 (2014). p. 1–28.

[2] Franceschi C Bonafe M Valensin S Olivieri F De Luca M Ottaviani E. Inflamm-aging. An evolutionary perspective on immunosenescence. Ann N Y Acad Sci. (2000) 908:244–54. 10.1111/j.1749-6632.2000.tb06651.x 10911963

[3] Hotamisligil GS. Inflammation and metabolic disorders. Nature (2006) 444:860–7. 10.1038/nature05485 17167474

[4] Miller RA. Aging and immune function: cellular and biochemical analyses. Exp Gerontol. (1994) 29:21–35. 8187838 10.1016/0531-5565(94)90060-4

[5] Palmer AK Kirkland JL. Aging and adipose tissue: potential interventions for diabetes and regenerative medicine. Exp Gerontol. (2016) 86:97–105. 10.1016/j.exger.2016.02.013 26924669 PMC5001933

[6] Gruver AL Hudson LL Sempowski GD. Immunosenescence of ageing. J Pathol. (2007) 211:144–56. 10.1002/path.2104 17200946 PMC1931833

[7] Michaud M Balardy L Moulis G Gaudin C Peyrot C Vellas B. Proinflammatory cytokines, aging, and age-related diseases. J Am Med Direct Assoc. (2013) 14:877–82. 10.1016/j.jamda.2013.05.009 23792036

[8] Nikolich-Zugich J. Aging of the T cell compartment in mice and humans: from no naive expectations to foggy memories. J Immunol. (2014) 193:2622–9. 10.4049/jimmunol.1401174 25193936 PMC4157314

[9] Blackman MA Woodland DL. The narrowing of the CD8 T cell repertoire in old age. Curr Opin Immunol. (2011) 23:537–42. 10.1016/j.coi.2011.05.005 21652194 PMC3163762

[10] Linton P-J Thoman ML. T cell senescence. Front. Biosci. (2001) 6:d248–61. 11171551 10.2741/linton

[11] Horber FF Gruber B Thomi F Jensen EX Jaeger P. Effect of sex and age on bone mass, body composition and fuel metabolism in humans. Nutrition (1997) 13:524–34. 10.1016/S0899-9007(97)00031-2 9263233

[12] Pascot A Lemieux S Lemieux I Prud'homme D Tremblay A Bouchard C. Age-related increase in visceral adipose tissue and body fat and the metabolic risk profile of premenopausal women. Diabetes Care (1999) 22:1471–8. 10480511 10.2337/diacare.22.9.1471

[13] Mraz M Haluzik M. The role of adipose tissue immune cells in obesity and low-grade inflammation. J Endocrinol. (2014) 222:R113–27. 10.1530/JOE-14-0283 25006217

[14] Tchkonia T Morbeck TE von Zglinicki T Van Deursen J Lustgarten J Scrable H. Fat tissue, aging, and cellular senescence. Aging Cell 9 (2010) 9:667–84. 10.1111/j.1474-9726.2010.00608.x 20701600 PMC2941545

[15] Mau T Yung R. Adipose tissue inflammation in aging. Exp Gerontol. (2018) 105:27–31. 10.1016/j.exger.2017.10.014 29054535 PMC5869077

[16] Wang W Seale P. Control of brown and beige fat development. Nat Rev Mol Cell Biol. (2016) 17:691–702. 10.1038/nrm.2016.96 27552974 PMC5627770

[17] Villarroya F Cereijo R Villarroya J Giralt M. Brown adipose tissue as a secretory organ. Nature Rev Endocrinol. (2017) 13:26–35. 10.1038/nrendo.2016.136 27616452

[18] Freedland ES. Role of a critical visceral adipose tissue threshold (CVATT) in metabolic syndrome: implications for controlling dietary carbohydrates: a review. Nutr Metab. (2004) 1:12. 10.1186/1743-7075-1-12 15530168 PMC535537

[19] Jensen MD. Role of body fat distribution and the metabolic complications of obesity. J Clin Endocrinol Metab. (2008) 93:S57–63. 10.1210/jc.2008-1585 18987271 PMC2585758

[20] Kuk JL Saunders TJ Davidson LE Ross R. Age-related changes in total and regional fat distribution. Ageing Res Rev. (2009) 8:339–48. 10.1016/j.arr.2009.06.001 19576300

[21] Nosalski R Guzik TJ. Perivascular adipose tissue inflammation in vascular disease. Br J Pharmacol. (2017) 174:3496–513. 10.1111/bph.13705 28063251 PMC5610164

[22] Olefsky JM Glass CK. Macrophages, inflammation, and insulin resistance. Ann Rev Physiol. (2010) 72:219–46. 10.1146/annurev-physiol-021909-135846 20148674

[23] Bapat SP Myoung Suh J Fang S Liu S Zhang Y Cheng A. Depletion of fat-resident Treg cells prevents age-associated insulin resistance. Nature (2015) 528:137–41. 26580014 10.1038/nature16151 PMC4670283

[24] Wu H Ghosh S Perrard XD Feng L Garcia GE Perrard JL. T-cell accumulation and regulated on activation, normal T cell expressed and secreted upregulation in adipose tissue in obesity. Circulation (2007) 115:1029–38. 10.1161/CIRCULATIONAHA.106.638379 17296858

[25] Wu H Perrard XD Wang Q Perrard JL Polsani VR Jones PH. CD11c expression in adipose tissue and blood and its role in diet-induced obesity. Arterioscler Thromb Vasc Biol. (2010) 30:186–92. 10.1161/ATVBAHA.109.198044 19910635 PMC2830649

[26] Weisberg SP McCann D Desai M Rosenbaum M Leibel RL Ferrante AW Jr. Obesity is associated with macrophage accumulation in adipose tissue. J Clin Investig. (2003) 112:1796–808. 10.1172/JCI200319246 14679176 PMC296995

[27] Xu H Barnes GT Yang Q Tan G Yang D Chou CJ. Chronic inflammation in fat plays a crucial role in the development of obesity-related insulin resistance. J Clin Investig. (2003) 112:1821–30. 10.1172/JCI200319451 14679177 PMC296998

[28] Lumeng CN Bodzin JL Saltiel AR. Obesity induces a phenotypic switch in adipose tissue macrophage polarization. J Clin Invest. (2007) 117:175–84. 10.1172/JCI29881 17200717 PMC1716210

[29] Morris DL Cho KW Delproposto JL Oatmen KE Geletka LM Martinez-Santibanez G. Adipose tissue macrophages function as antigen-presenting cells and regulate adipose tissue CD4+ T cells in mice. Diabetes (2013) 62:2762–72. 10.2337/db12-1404 23493569 PMC3717880

[30] Feuerer M Herrero L Cipolletta D Naaz A Wong J Nayer A Lean, but not obese, fat is enriched for a unique population of regulatory T cells that affect metabolic parameters. Nature Med. (2009) 15:930–9. 10.1038/nm.2002 19633656 PMC3115752

[31] Nishimura S Manabe I Nagasaki M Eto K Yamashita H Ohsugi M. CD8+ effector T cells contribute to macrophage recruitment and adipose tissue inflammation in obesity. Nat Med. (2009) 15:914–20. 10.1038/nm.1964 19633658

[32] Winer S Chan Y Paltser G Truong D Tsui H Bahrami J. Normalization of obesity-associated insulin resistance through immunotherapy. Nat Med. (2009) 15:921–9. 10.1038/nm.2001 19633657 PMC3063199

[33] McLaughlin T Liu LF Lamendola C Shen L Morton J Rivas H. T-cell profile in adipose tissue is associated with insulin resistance and systemic inflammation in humans. Arterioscler Thromb Vasc Biol. (2014) 34:2637–43. 10.1161/ATVBAHA.114.304636 25341798 PMC4445971

[34] Garg SK Delaney C Shi H Yung R. Changes in adipose tissue macrophages and T cells during aging. Crit. Rev. Immunol. (2014) 34:1–14. 10.1615/CritRevImmunol.2013006833 24579699 PMC3942798

[35] Nagelkerken L Hertogh-Huijbregts A Dobber R Drager A. Age-related changes in lymphokine production related to a decreased number of CD45RBhi CD4+ T cells. Eur J Immunol. (1991) 21:273–81. 10.1002/eji.1830210206 1671835

[36] Johnson TE. Recent results: biomarkers of aging. Exp Gerontol. (2006) 41:1243–6. 10.1016/j.exger.2006.09.006 17071038

[37] O'Mahony L Holland J Jackson J Feighery C Hennessy TP Mealy K. Quantitative intracellular cytokine measurement: age-related changes in proinflammatory cytokine production. Clin Exp Immunol. (1998) 113:213–9. 10.1046/j.1365-2249.1998.00641.x 9717970 PMC1905038

[38] Zanni F Vescovini R Biasini C Fagnoni F Zanlari L Telera A. Marked increase with age of type 1 cytokines within memory and effector/cytotoxic CD8+ T cells in humans: a contribution to understand the relationship between inflammation and immunosenescence. Exp Gerontol. (2003) 38:981–7. 10.1016/S0531-5565(03)00160-8 12954485

[39] Trott DW Henson GD Ho MH Allison SA Lesniewski LA Donato AJ Age-related arterial immune cell infiltration in mice is attenuated by caloric restriction or voluntary exercise. Exp Gerontol. (2016) 109:99–107. 10.1016/j.exger.2016.12.016 28012941 PMC5481497

[40] Mo R Chen J Han Y Bueno-Cannizares C Misek DE Lescure PA. T cell chemokine receptor expression in aging. J Immunol. (2003) 170:895–904. 10.4049/jimmunol.170.2.895 12517955

[41] Cane S Ponnappan S Ponnappan U. Altered regulation of CXCR4 expression during aging contributes to increased CXCL12-dependent chemotactic migration of CD4(+) T cells. Aging Cell (2012) 11:651–8. 10.1111/j.1474-9726.2012.00830.x 22568557 PMC3399962

[42] Yung R Mo R Grolleau-Julius A Hoeltzel M. The effect of aging and caloric restriction on murine CD8+ T cell chemokine receptor gene expression. Immun Ageing (2007) 4:8. 10.1186/1742-4933-4-8 18001471 PMC2200663

[43] Lumeng CN Liu J Geletka L Delaney C Delproposto J Desai A. Aging is associated with an increase in T cells and inflammatory macrophages in visceral adipose tissue. J Immunol. (2011) 187:6208–16. 10.4049/jimmunol.1102188 22075699 PMC3237772

[44] Jiang E Perrard XD Yang D Khan IM Perrard JL Smith CW. Essential role of CD11a in CD8+ T-cell accumulation and activation in adipose tissue. Arterioscler Thromb Vasc Biol. (2014) 34:34–43. 10.1161/ATVBAHA.113.302077 24158516 PMC4060534

[45] Ahnstedt H Roy-O'Reilly M Spychala MS Mobley AS Bravo-Alegria J Chauhan A. Sex differences in adipose tissue CD8(+) T cells and regulatory T cells in middle-aged mice. Front Immunol. (2018) 9:659. 10.3389/fimmu.2018.00659 29670627 PMC5893719

[46] Krishna KB Stefanovic-Racic M Dedousis N Sipula I O'Doherty RM. Similar degrees of obesity induced by diet or aging cause strikingly different immunologic and metabolic outcomes. Physiol Rep. (2016) 4:e12708. 10.14814/phy2.12708 27033445 PMC4814885

[47] Lu L Barbi J Pan F. The regulation of immune tolerance by FOXP3. Nat Rev Immunol. (2017) 17:703–17. 10.1038/nri.2017.75 28757603 PMC5793224

[48] Sakaguchi S Yamaguchi T Nomura T Ono M. Regulatory T cells and immune tolerance. Cell (2008) 133:775–87. 10.1016/j.cell.2008.05.009 18510923

[49] Hu X Ivashkiv LB. Cross-regulation of signaling pathways by interferon-gamma: implications for immune responses and autoimmune diseases. Immunity (2009) 31:539–50. 10.1016/j.immuni.2009.09.002 19833085 PMC2774226

[50] Cipolletta D Cohen P Spiegelman BM Benoist C Mathis D. Appearance and disappearance of the mRNA signature characteristic of Treg cells in visceral adipose tissue: age, diet, and PPARγ effects. Proc Natl Acad Sci USA. (2015) 112:482–87. 10.1073/pnas.1423486112 25550516 PMC4299242

[51] Kohlgruber AC Gal-Oz ST LaMarche NM Shimazaki M Duquette D Nguyen HN. gammadelta T cells producing interleukin-17A regulate adipose regulatory T cell homeostasis and thermogenesis. Nat Immunol. (2018) 19:464–74. 10.1038/s41590-018-0094-2 29670241 PMC8299914

[52] Mehta P Nuotio-Antar AM Smith CW. gammadelta T cells promote inflammation and insulin resistance during high fat diet-induced obesity in mice. J Leukoc Biol. (2015) 97:121–34. 10.1189/jlb.3A0414-211RR 25395302 PMC4377824

[53] Lynch L Nowak M Varghese B Clark J Hogan AE Toxavidis V. Adipose tissue invariant NKT cells protect against diet-induced obesity and metabolic disorder through regulatory cytokine production. Immunity (2012) 37:574–87. 10.1016/j.immuni.2012.06.016 22981538 PMC4991771

[54] Subramanian S Turner MS Ding Y Goodspeed L Wang S Buckner JH. Increased levels of invariant natural killer T lymphocytes worsen metabolic abnormalities and atherosclerosis in obese mice. J Lipid Res. (2013) 54:2831–41. 10.1194/jlr.M041020 23922382 PMC3770095

[55] Lopez S Garcia-Serrano S Gutierrez-Repiso C Rodriguez-Pacheco F Ho-Plagaro A Santiago-Fernandez C. Tissue-specific phenotype and activation of iNKT cells in morbidly obese subjects: interaction with adipocytes and effect of bariatric surgery. Obes Surg. (2018) 28:2774–82. 10.1007/s11695-018-3215-y 29619756

[56] Wu D Molofsky AB Liang HE Ricardo-Gonzalez RR Jouihan HA Bando JK. Eosinophils sustain adipose alternatively activated macrophages associated with glucose homeostasis. Science (2011) 332:243–7. 10.1126/science.1201475 21436399 PMC3144160

[57] Bouloumie A Casteilla L Lafontan M. Adipose tissue lymphocytes and macrophages in obesity and insulin resistance: makers or markers, and which comes first? Arterioscler Thromb Vasc Biol. (2008) 28:1211–3. 10.1161/ATVBAHA.108.168229 18565843

[58] Deng T Lyon CJ Minze LJ Lin J Zou J Liu JZ. Class II major histocompatibility complex plays an essential role in obesity-induced adipose inflammation. Cell Metab. (2013) 17:411–22. 10.1016/j.cmet.2013.02.009 23473035 PMC3619392

[59] Khan IM Dai Perrard XY Perrard JL Mansoori A Smith CW Wu H. Attenuated adipose tissue and skeletal muscle inflammation in obese mice with combined CD4+ and CD8+ T cell deficiency. Atherosclerosis (2014) 233:419–28. 10.1016/j.atherosclerosis.2014.01.011 24530773 PMC4094239

[60] Ilan Y Maron R Tukpah AM Maioli TU Murugaiyan G Yang K. Induction of regulatory T cells decreases adipose inflammation and alleviates insulin resistance in ob/ob mice. Proc Natl Acad Sci USA. (2010) 107:9765–70. 10.1073/pnas.0908771107 20445103 PMC2906892

[61] Park YJ Park J Huh JY Hwang I Choe SS Kim JB. Regulatory roles of invariant natural killer T cells in adipose tissue inflammation: defenders against obesity-induced metabolic complications. Front Immunol. (2018) 9:1311. 10.3389/fimmu.2018.01311 29951059 PMC6008523

[62] Ren Y Sekine-Kondo E Tateyama M Kasetthat T Wongratanacheewin S Watarai H. New genetically manipulated mice provide insights into the development and physiological functions of invariant natural killer T cells. Front Immunol. (2018) 9:1294. 10.3389/fimmu.2018.01294 29963043 PMC6010523

[63] Satoh M Iwabuchi K. Role of natural killer T cells in the development of obesity and insulin resistance: insights from recent progress. Front Immunol. (2018) 9:1314. 10.3389/fimmu.2018.01314 29942311 PMC6004523

[64] Wu H Ballantyne CM. Skeletal muscle inflammation and insulin resistance in obesity. J Clin Invest. (2017) 127:43–54. 10.1172/JCI88880 28045398 PMC5199705

[65] Khan IM Perrard XY Brunner G Lui H Sparks LM Smith SR. Intermuscular and perimuscular fat expansion in obesity correlates with skeletal muscle T cell and macrophage infiltration and insulin resistance. Int J Obes. (2015) 39:1607–18. 10.1038/ijo.2015.104 26041698 PMC5007876

[66] Sun K Kusminski CM Scherer PE. Adipose tissue remodeling and obesity. J Clin Investig. (2011) 121:2094–101. 10.1172/JCI45887 21633177 PMC3104761

[67] Lumeng CN Deyoung SM Bodzin JL Saltiel AR. Increased inflammatory properties of adipose tissue macrophages recruited during diet-induced obesity. Diabetes (2007) 56:16–23. 10.2337/db06-1076 17192460

[68] Cho KW Morris DL DelProposto JL Geletka L Zamarron B Martinez-Santibanez G. An MHC II-dependent activation loop between adipose tissue macrophages and CD4+ T cells controls obesity-induced inflammation. Cell Rep. (2014) 9:605–17. 10.1016/j.celrep.2014.09.004 25310975 PMC4252867

[69] Deng T Liu J Deng Y Minze L Xiao X Wright V Adipocyte adaptive immunity mediates diet-induced adipose inflammation and insulin resistance by decreasing adipose Treg cells. Nature Commun. (2017) 8:15725 10.1038/ncomms15725

[70] McGillicuddy FC Chiquoine EH Hinkle CC Kim RJ Shah R Roche HM. Interferon gamma attenuates insulin signaling, lipid storage, and differentiation in human adipocytes via activation of the JAK/STAT pathway. J Biol Chem. (2009) 284:31936–44. 10.1074/jbc.M109.061655 19776010 PMC2797265

[71] Bluher M Fasshauer M Tonjes A Kratzsch J Schon MR Paschke R. Association of interleukin-6, C-reactive protein, interleukin-10 and adiponectin plasma concentrations with measures of obesity, insulin sensitivity and glucose metabolism. Exp Clin Endocrinol Diabetes (2005) 113:534–7. 10.1055/s-2005-872851 16235156

[72] Scarpelli D Cardellini M Andreozzi F Laratta E Hribal ML Marini MA Variants of the interleukin-10 promoter gene are associated with obesity and insulin resistance but not type 2 diabetes in caucasian italian subjects. Diabetes (2006) 55:1529–33. 10.2337/db06-0047 16644716

[73] SchererPE Adipose tissue: from lipid storage compartment to endocrine organ. Diabetes (2006) 55:1537–45. 10.2337/db06-0263 16731815

[74] Vosselman MJ van Marken Lichtenbelt WD Schrauwen P. Energy dissipation in brown adipose tissue: from mice to men. Mol Cell Endocrinol. (2013) 379:43–50. 10.1016/j.mce.2013.04.017 23632102

[75] Ikeda K Maretich P Kajimura S. The common and distinct features of brown and beige adipocytes. Trends Endocrinol Metab. (2018) 29:191–200. 10.1016/j.tem.2018.01.001 29366777 PMC5826798

[76] Villarroya F Cereijo R Villarroya J Gavalda-Navarro A Giralt M. Toward an understanding of how immune cells control brown and beige adipobiology. Cell Metab. (2018) 27:954–61. 10.1016/j.cmet.2018.04.006 29719233

[77] Qiu Y Nguyen KD Odegaard JI Cui X Tian X Locksley RM. Eosinophils and type 2 cytokine signaling in macrophages orchestrate development of functional beige fat. Cell (2014) 157:1292–308. 10.1016/j.cell.2014.03.066 24906148 PMC4129510

[78] Lee MW Odegaard JI Mukundan L Qiu Y Molofsky AB Nussbaum JC. Activated type 2 innate lymphoid cells regulate beige fat biogenesis. Cell (2015) 160:74–87. 10.1016/j.cell.2014.12.011 25543153 PMC4297518

[79] Moysidou M Karaliota S Kodela E Salagianni M Koutmani Y Katsouda A. CD8+ T cells in beige adipogenesis and energy homeostasis. JCI Insight (2018) 3:95456. 10.1172/jci.insight.95456 29515042 PMC5922290

[80] Molofsky AB Nussbaum JC Liang HE Van Dyken SJ Cheng LE Mohapatra A. Innate lymphoid type 2 cells sustain visceral adipose tissue eosinophils and alternatively activated macrophages. J Exp Med. (2013) 210:535–49. 10.1084/jem.20121964 23420878 PMC3600903

[81] Uhm M Saltiel AR. White, brown, and beige; type 2 immunity gets hot. Immunity (2015) 42:15–7. 10.1016/j.immuni.2015.01.001 25607455

[82] Zhu J. T helper 2 (Th2) cell differentiation, type 2 innate lymphoid cell (ILC2) development and regulation of interleukin-4 (IL-4) and IL-13 production. Cytokine (2015) 75:14–24. 10.1016/j.cyto.2015.05.010 26044597 PMC4532589

[83] Lee YH Petkova AP Mottillo EP Granneman JG. *In vivo* identification of bipotential adipocyte progenitors recruited by beta3-adrenoceptor activation and high-fat feeding. Cell Metab. (2012) 15:480–91. 10.1016/j.cmet.2012.03.009 22482730 PMC3322390

[84] Fischer K Ruiz HH Jhun K Finan B Oberlin DJ van der Heide V. Alternatively activated macrophages do not synthesize catecholamines or contribute to adipose tissue adaptive thermogenesis. Nat Med. (2017) 23:623–30. 10.1038/nm.4316 28414329 PMC5420449

[85] Lynch L Michelet X Zhang S Brennan PJ Moseman A Lester C. Regulatory iNKT cells lack expression of the transcription factor PLZF and control the homeostasis of T(reg) cells and macrophages in adipose tissue. Nat Immunol. (2015) 16:85–95. 10.1038/ni.3047 25436972 PMC4343194

[86] Lynch L Hogan AE Duquette D Lester C Banks A LeClair K. iNKT cells induce FGF21 for thermogenesis and are required for maximal weight loss in GLP1 therapy. Cell Metab. (2016) 24:510–19. 10.1016/j.cmet.2016.08.003 27593966 PMC5061124

[87] Fisher FM Kleiner S Douris N Fox EC Mepani RJ Verdeguer F. FGF21 regulates PGC-1alpha and browning of white adipose tissues in adaptive thermogenesis. Genes Dev. (2012) 26:271–81. 10.1101/gad.177857.111 22302939 PMC3278894

[88] Holland WL Adams AC Brozinick JT Bui HH Miyauchi Y Kusminski CM. An FGF21-adiponectin-ceramide axis controls energy expenditure and insulin action in mice. Cell Metab. (2013) 17:790–7. 10.1016/j.cmet.2013.03.019 23663742 PMC3667496

[89] Saito M Okamatsu-Ogura Y Matsushita M Watanabe K Yoneshiro T Nio-Kobayashi J. High incidence of metabolically active brown adipose tissue in healthy adult humans: effects of cold exposure and adiposity. Diabetes (2009) 58:1526–31. 10.2337/db09-0530 19401428 PMC2699872

[90] Rogers NH Landa A Park S Smith RG. Aging leads to a programmed loss of brown adipocytes in murine subcutaneous white adipose tissue. Aging Cell (2012) 11:1074–83. 10.1111/acel.12010 23020201 PMC3839316

[91] Berry DC Jiang Y Arpke RW Close EL Uchida A Reading D Cellular aging contributes to failure of cold-induced beige adipocyte formation in old mice and humans. Cell Metab. (2017) 25:166–81. 10.1016/j.cmet.2016.10.023 27889388 PMC5226893

[92] Kolodin D van Panhuys N Li C Magnuson AM Cipolletta D Miller CM. Antigen- and cytokine-driven accumulation of regulatory T cells in visceral adipose tissue of lean mice. Cell Metab. (2015) 21:543–57. 10.1016/j.cmet.2015.03.005 25863247 PMC4747251

[93] Li C DiSpirito JR Zemmour D Spallanzani RG Kuswanto W Benoist C. TCR transgenic mice reveal stepwise, multi-site acquisition of the distinctive fat-treg phenotype. Cell (2018) 174:285–99.e12. 10.1016/j.cell.2018.05.004 29887374 PMC6046274

[94] Vasanthakumar A Moro K Xin A Liao Y Gloury R Kawamoto S The transcriptional regulators IRF4, BATF and IL-33 orchestrate development and maintenance of adipose tissue-resident regulatory T cells. Nat Immunol. (2015) 16:276–85. 10.1038/ni.3085 25599561

[95] Cipolletta D Feuerer M Li A Kamei N Lee J Shoelson SE. PPAR-gamma is a major driver of the accumulation and phenotype of adipose tissue Treg cells. Nature (2012) 486:549–53. 10.1038/nature11132 22722857 PMC3387339

[96] Suganami T Nishida J Ogawa Y. A paracrine loop between adipocytes and macrophages aggravates inflammatory changes: role of free fatty acids and tumor necrosis factor alpha. Arterioscler Thromb Vasc Biol. (2005) 25:2062–8. 10.1161/01.ATV.0000183883.72263.13 16123319

[97] Lu H Smith CW Perrard J Bullard D Tang L Shappell SB. LFA-1 is sufficient in mediating neutrophil emigration in Mac-1-deficient mice. J Clin Investig. (1997) 99:1340–50. 10.1172/JCI119293 9077544 PMC507950

[98] Pribila JT Quale AC Mueller KL Shimizu Y. Integrins and T cell-mediated immunity. Ann Rev Immunol. (2004) 22:157–80. 10.1146/annurev.immunol.22.012703.104649 15032577

[99] Carman CV Martinelli R. T Lymphocyte-endothelial interactions: emerging understanding of trafficking and antigen-specific immunity. Front Immunol. (2015) 6:603. 10.3389/fimmu.2015.00603 26635815 PMC4657048

[100] Duffaut C Zakaroff-Girard A Bourlier V Decaunes P Maumus M Chiotasso P. Interplay between human adipocytes and T lymphocytes in obesity: CCL20 as an adipochemokine and T lymphocytes as lipogenic modulators. Arterioscler Thromb Vasc Biol. (2009) 29:1608–14. 10.1161/ATVBAHA.109.192583 19644053

[101] Kintscher U Hartge M Hess K Foryst-Ludwig A Clemenz M Wabitsch M.. T-lymphocyte infiltration in visceral adipose tissue: a primary event in adipose tissue inflammation and the development of obesity-mediated insulin resistance. Arterioscler Thromb Vasc Biol. (2008) 28:1304–10. 10.1161/ATVBAHA.108.165100 18420999

[102] Hennecke J Wiley DC. T cell receptor-MHC interactions up close. Cell (2001) 104:1–4. 10.1016/S0092-8674(01)00185-4 11163234

[103] Xiao L Yang X Lin Y Li S Jiang J Qian S. Large adipocytes function as antigen-presenting cells to activate CD4(+) T cells via upregulating MHCII in obesity. Int J Obes. (2016) 40:112–20. 10.1038/ijo.2015.145 26248660 PMC4722243

[104] Reith W LeibundGut-Landmann S Waldburger JM. Regulation of MHC class II gene expression by the class II transactivator. Nat Rev Immunol. (2005) 5:793–806. 10.1038/nri1708 16200082

[105] Morris DL Oatmen KE Mergian TA Cho KW DelProposto JL Singer K. CD40 promotes MHC class II expression on adipose tissue macrophages and regulates adipose tissue CD4+ T cells with obesity. J Leukoc Biol. (2016) 99:1107–19. 10.1189/jlb.3A0115-009R 26658005 PMC4952010

[106] Shirakawa K Yan X Shinmura K Endo J Kataoka M Katsumata Y. Obesity accelerates T cell senescence in murine visceral adipose tissue. J Clin Investig. (2016) 126:4626–39. 10.1172/JCI88606 27820698 PMC5127667

[107] Berg RE Cordes CJ Forman J. Contribution of CD8+ T cells to innate immunity: IFN-gamma secretion induced by IL-12 and IL-18. Eur J Immunol. (2002) 32:2807–16. 10.1002/1521-4141(2002010)32:10<2807::AID-IMMU2807>3.0.CO;2-0 12355433

[108] Soudja SM Ruiz AL Marie JC Lauvau G. Inflammatory monocytes activate memory CD8(+) T and innate NK lymphocytes independent of cognate antigen during microbial pathogen invasion. Immunity (2012) 37:549–62. 10.1016/j.immuni.2012.05.029 22940097 PMC3456987

[109] Larsen CM Faulenbach M Vaag A Vølund A Ehses JA Seifert B. Interleukin-1–receptor antagonist in type 2 diabetes mellitus. N Engl J Med. (2007) 356:1517–26. 10.1056/NEJMoa065213 17429083

[110] Larsen CM Faulenbach M Vaag A Ehses JA Donath MY Mandrup-Poulsen T. Sustained effects of interleukin-1 receptor antagonist treatment in type 2 diabetes. Diabetes Care (2009) 32:1663–8. 10.2337/dc09-0533 19542207 PMC2732140

[111] Brosius FC Tuttle KR Kretzler M. JAK inhibition in the treatment of diabetic kidney disease. Diabetologia (2016) 59:1624–7. 10.1007/s00125-016-4021-5 27333885 PMC4942738

[112] Tuttle KR Brosius FC III Adler SG Kretzler M Mehta RL Tumlin JA. JAK1/JAK2 inhibition by baricitinib in diabetic kidney disease: results from a Phase 2 randomized controlled clinical trial. Nephrol Dial Transplant. (2018) 33:1950–9. 10.1093/ndt/gfx377 29481660 PMC6212720

[113] Friedman SL Ratziu V Harrison SA Abdelmalek MF Aithal GP Caballeria J. A randomized, placebo-controlled trial of cenicriviroc for treatment of nonalcoholic steatohepatitis with fibrosis. Hepatology (2018) 67:1754–67. 10.1002/hep.29477 28833331 PMC5947654

[114] Krenkel O Puengel T Govaere O Abdallah AT Mossanen JC Kohlhepp M. Therapeutic inhibition of inflammatory monocyte recruitment reduces steatohepatitis and liver fibrosis. Hepatology (2018) 67:1270–83. 10.1002/hep.29544 28940700

